# l-Arginine and B vitamins improve endothelial function in subjects with mild to moderate blood pressure elevation

**DOI:** 10.1007/s00394-016-1342-6

**Published:** 2016-11-05

**Authors:** Daniel Menzel, Hermann Haller, Manfred Wilhelm, Horst Robenek

**Affiliations:** 1 Stuttgart, Germany; 2 Department of Nephrology and Hypertension Medicine, Hannover Medical School, University of Hannover, Hannover, Germany; 3 Ulm University of Applied Sciences, Ulm, Germany; 4 University Clinic Münster, Albert-Schweitzer-Campus 1, Domagkstr. 3, 48149 Münster, Germany

**Keywords:** Endothelial function, Atherosclerosis, Blood pressure, l-Arginine, B vitamins

## Abstract

**Purpose:**

The aim of this trial was to investigate the influence of a dietetic product consisting of a unique combination of l-arginine with the vitamins B_6_, folic acid and B_12_ (Telcor^®^ Arginin plus) on endothelial dysfunction.

**Methods:**

Subjects aged 40–65 years with mild to moderate blood pressure (BP) elevation not treated with anti-hypertensive drugs were randomly assigned to either the dietetic product (*n* = 40) or a matching placebo (*n* = 41) for 3 months with open follow-up for a further 3 months. Postprandial change in endothelial function was assessed using the validated reactive hyperaemia index (RHI) at 3 months compared to the study onset (RHI post–pre, visit 3–visit 1; ΔΔRHI). Secondary parameters included BP and plasma homocysteine concentration.

**Results:**

The primary efficacy analysis revealed superiority of the nutritional intervention over placebo (*p* = 0.0349) in reducing the deterioration of endothelial function. While in the active group ΔΔRHI increased (0.371 ± 0.122), almost no change could be detected in the placebo group (0.031 ± 0.100), thus demonstrating a significant improvement in vascular function in the intervention group. Moreover, the intervention reduced BP and homocysteine levels. Non-serious adverse events were equally distributed in both groups, and none of the events were assessed as possibly intervention-related by the investigators.

**Conclusions:**

This trial confirmed the effective and safe use of dietary management with l-arginine in combination with B vitamins. The primary efficacy analysis demonstrated a statistically significant superiority of the combination of l-arginine with B vitamins over placebo in improving and restoring impaired endothelial function and lowering BP in patients with mild to moderate blood pressure elevation.

## Introduction

Mediated by nitric oxide (NO), intact endothelial cells control vascular homoeostasis by regulating vascular tone and preventing smooth muscle cell proliferation, monocyte adhesion to the endothelium and platelet aggregation, thereby protecting blood vessels from the formation of atherosclerosis [1]. Endothelial dysfunction (ED) is characterized by a reduced bioavailability of NO as well as an impairment of endothelium-dependent vasodilatation [2–4]. NO is formed from the semi-essential amino acid l-arginine by the endothelial nitric oxide synthase (eNOS) enzyme as long as the B vitamins B_6_, folic acid and B_12_ are provided at sufficient levels [5]. NO is involved in numerous regulatory mechanisms of the vascular system, and lack of NO is now widely regarded as a key molecular mechanism causing cardiovascular disease [4, 6–8]. Atherosclerotic cardiovascular diseases are the leading cause of morbidity and mortality [9].

Atherosclerosis is initiated by the response of vascular endothelial cells to injury caused by cardiovascular risk factors such as essential hypertension, several forms of dyslipidemia, diabetes mellitus, cigarette smoking, ageing, hyperhomocysteinemia, obesity and lack of physical activity [4]. Continuous exposure to various risk factors promotes plaque progression and destabilization which ultimately results in plaque rupture and an acute thrombotic occlusion of one or more coronary or brain arteries, causing stroke or myocardial infarction. The vascular endothelium serves as a barrier separating the vessel wall from the blood and as such constitutes a primary sensitive target for the damaging effects of atherogenic risk factors.

NO is a potent endogenous vasodilator and inhibits aggregation of platelets, adhesion of monocytes to the endothelium and smooth muscle cell proliferation [1–3, 5, 10]. Together, these functions make NO a significant endogenous anti-atherosclerotic mediator.

One mechanism that explains the occurrence of ED and the lack of NO is the presence of elevated blood levels of asymmetric dimethylarginine (ADMA), an l-arginine analogue, in patients with cardiovascular diseases [3, 11–15]. ADMA is an endogenous inhibitor of the NO synthase that inhibits NO formation, and in this way it can also impair vascular function. ADMA displaces l-arginine from its binding sites that enable NO formation, binding to them with a tenfold higher affinity [11, 16, 17].

Cardiovascular diseases are associated with low l-arginine levels and a high ADMA: l-arginine ratio that reduces the bioavailability of NO [15, 18]. This imbalance is not only facilitated further by the inadequate synthesis and increased degradation of NO, but also by an increased loss of the substrate l-arginine. The availability of l-arginine can further be affected by the fact that 50–70% of dietary l-arginine is metabolized by the intestinal mucosa and does not even enter the circulation [19]. Studies have shown that dietary supplementation with l-arginine efficiently reverses the ED in patients with high ADMA levels and augments NO production [17]. In addition, l-arginine supplementation results in enhanced inhibition of platelet aggregation and monocyte adhesion and reduces vascular smooth muscle cell proliferation [20].

With advanced age, the physiological demand for l-arginine increases considerably [11, 12, 17]. Ageing is often associated with cardiovascular disease since ADMA levels are increased fourfold during advanced age [17].

Although some studies showed mixed results as regards the effects of an l-arginine supplementation on cardiovascular parameters (which may have been due to patient selection, dosing regimens and individual variation [21]), the majority of more recent studies show that supplementation with l-arginine restores vascular function and improves the clinical symptoms of various diseases associated with vascular dysfunction [22–27]. A widely criticized study was published by Schulman and colleagues in 2006 [28], allegedly showing a higher rate of fatalities in subjects with acute ST-segment elevation myocardial infarction and who had received 9 g l-arginine per day. The study, which was not powered for mortality, was stopped early and considered unreliable in the following years due to relevant study limitations [29, 30]. So, in a review by Shao and Hathcock and a letter to the editor by Abumrad and Barbul, the authors state that the outcome is inconsistent with the body of literature and appears to be a random anomaly [29, 30]. In numerous clinical studies, the administration of l-arginine showed promising effects in patients with cardiovascular and other diseases, and such effects have been confirmed by meta-analyses and recent systematic reviews [5, 22–25, 31–33].

Further studies have shown that a normalization of blood flow and blood pressure (BP) as well as a reduction in homocysteine levels can be achieved by dietary administration of l-arginine and sufficient amounts of B vitamins [4, 5, 17, 22, 23, 34, 35], even at low doses of 3 g per day [16, 22, 23]. l-arginine and B vitamins are particularly apt at restoring endothelial health and reversing vascular degeneration and do this by stimulating nitric oxide formation [5, 17]. In the study presented here, our intention was to confirm this using a special dietary combination which was characterized by low amounts of l-arginine, vitamin B_6_, folic acid and vitamin B_12_. An editorial published in Cardiovascular Research on l-arginine and B vitamin supplementation in 2012 further encouraged this investigation reporting on their potent synergistic vasoprotective effects [36]. The study was a controlled trial investigating the efficacy, safety and tolerability of a specific combination of l-arginine and B vitamins (intervention) and particularly its influence on ED and associated diseases such as hypertension and hyperhomocysteinemia in subjects with mild to moderately elevated BP. Since the induction of ED by provision of a fatty meal [37] can be considered as a recognized method for clinical trials, and since reactive hyperaemia peripheral arterial tonometry (RHI) adequately depicts the overall health of the endothelium [38, 39], both methods were chosen to evaluate the endothelial function before and after dietary intervention.

## Methods

The controlled interventional trial was performed as a confirmatory, prospective, randomized, placebo-controlled, double-blind, single-centre study.

*Intervention products* Telcor^®^ Arginin plus (TAP); 2 capsules b.i.d.; total daily amounts: 2.4 g l-arginine, 3 mg vitamin B_6_, 0.4 mg folic acid, 2 µg vitamin B_12_ (intervention) or a matching placebo with the same visual appearance containing of microcrystalline cellulose.

*Subjects* 81 eligible subjects aged 40–65 years (age 53.8 ± 5.8 years, BMI 25.3 ± 2.7 kg/m^2^, 37% female, all non-smokers) were randomly assigned to receive either the nutritional intervention (*n* = 40) or a matching placebo (*n* = 41) for 3 months. During the subsequent follow-up phase, all subjects received TAP intervention for 3 months.

Subjects had to be either male or female (postmenopausal) aged 40–65 years with slightly to moderately elevated BP (130 to ≤149 mmHg SBP) and had to be under assessment for other risk factors (end organ damage, diabetes, clinically relevant cardiovascular diseases) and not receiving and not requiring any antihypertensive drug therapy (defined by the German hypertension guideline/Deutsche Hochdruckliga). Moreover, subjects characterized by severe cardiovascular diseases, obesity (BMI >30 kg/m^2^), interfering medication (e.g. anti-hypertensives, PDE-5 inhibitors) or sleep apnoea were excluded. They also had to be non-smokers. Participants were asked to maintain their usual diet, medications and lifestyle during the entire course of the study. Alcohol consumption and physical activity 24 h before the measurements were prohibited. Lifestyle changes were evaluated by using questionnaires at visits 3 and 5.

**Fig. 1:**
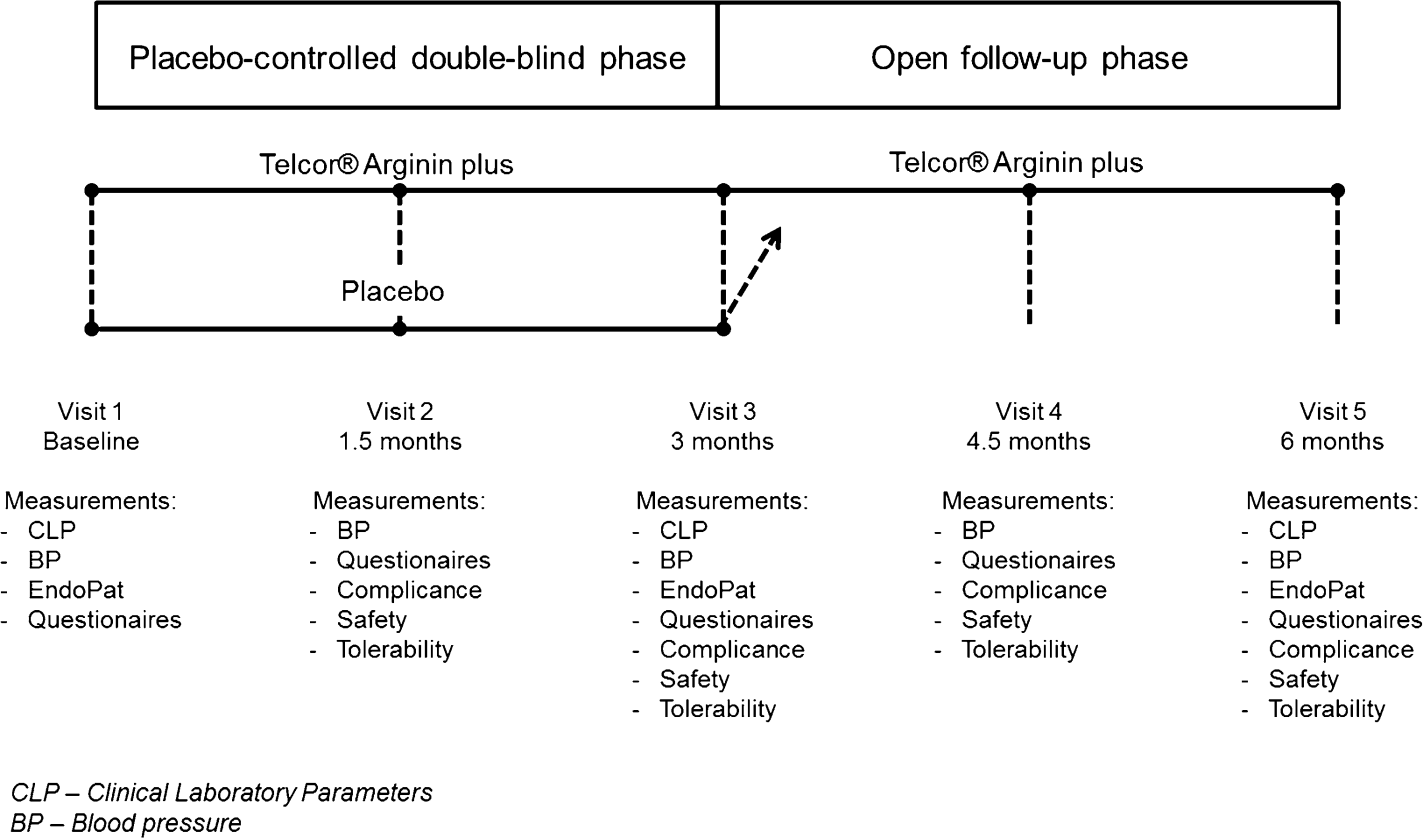
Trial design

*Overall trial design* This controlled interventional trial was performed as a monocentric, randomized, placebo-controlled, double-blind, parallel group trial for the first 3 months in phase I which was then followed by an open 3-month follow-up phase during which the intervention was given to all participants in phase II (Fig. 1). Randomization was made using a computer-generated randomization list. Moreover, randomization was stratified by gender. The compliance as assessed by counting capsules usage exceeded 95% in both study phases. Trial registration: German Clinical Trials Register No.: DRKS00010276.

*Trial Endpoints* The primary endpoint was defined as the postprandial (fatty meal, FM-induced) change in endothelial function assessed by means of the validated reactive hyperaemia index (RHI) at 3 months compared with the study onset (RHI post–pre, visit 3–visit 1; the ΔΔRHI). The primary efficacy analysis focused on the difference between intervention and placebo for assessing the possible impact of l-arginine and B vitamins in preventing FM-induced ED. Secondary efficacy parameters included changes in BP and homocysteine levels. All parameters were monitored over the entire study period of 6 months. Compliance, safety and tolerability of the intervention were determined by questionnaires, physical examinations, electrocardiography and routine clinical and laboratory parameters.

*Assessment of endothelial function* Normal physiological functioning of the endothelium was assessed by reactive hyperaemia peripheral arterial tonometry using the non-invasive EndoPAT™ (Itamar Medical Inc., Franklin, MA, USA) procedure [40]. The EndoPAT system is based on peripheral arterial tone (PAT) signal technology, a non-invasive plethysmographic method measuring pulsatile volume changes in the digital bed. The user-independent test was performed in a temperature-controlled and quiet environment. The test quantifies endothelium-mediated changes in vascular tone elicited by a 5-min occlusion of the brachial artery using a standard BP cuff inflated to a suprasystolic pressure. When the cuff was deflated, the surge of blood flow caused an endothelium-dependent, flow-mediated dilatation leading to reactive hyperaemia and an increase in the PAT signal amplitude. Measurements from the contralateral probe were used to control for non-endothelial-dependent changes in vascular tone. The postocclusion-to-preocclusion ratio, called the EndoScore or the reactive hyperaemia index (RHI), was calculated using the EndoPAT™ 2000 software [41]. A normal RHI score is defined as 1.67 or higher while lower values indicate vascular dysfunction which is related to an impairment of endothelium-dependent vasodilatation, and which is considered to be an independent predictor of cardiovascular morbidity and mortality [42, 43].

Impairment of endothelial function was induced by a fatty meal consisting of 30% cream at 3 ml/kg. Baseline measurements were conducted in the morning after a standardized meal in the evening before. TAP was administered at least 8–12 h before baseline and 30 min before intake of fatty meal at visits V3 and V5.

*Homocysteine* Plasma homocysteine levels were determined using an S-Monovette^®^ with 2.7 ml HCY/Z-Gel (Sarstedt, Nümbrecht, Germany) and HPLC.

**Blood Pressure**: *Home BP monitoring*: 7 days before each visit, the volunteers performed daily BP measurements at home using a standardized device (Boso^®^ medicus PC 2). Data were collected each day in the morning. Every measurement was documented along with the time and date. The mean of these 7 days for systolic and diastolic BP was used for evaluating the BP. *Ambulatory blood pressure monitoring (ABPM)*: BP readings were performed on the day of visit on an ambulatory basis over 24 h by using a standardized monitoring device (Boso^®^ TM-2430 PC 2) which collected data (systolic pressure, diastolic pressure, heart rate and mean arterial pressure) every 15 min during the day and every 30 min during the night.

*Statistics* Appropriate statistical analyses were performed using GraphPad Prism version 5.0 (GraphPad Software Inc., La Jolla, CA, USA). All parameters were analysed on an intention-to-treat (ITT) basis by descriptive and inferential statistics. Based on available empirical evidence [44, 45], the calculated sample size was 37 for each group (alpha 0.05, power 0.80). Data distributions were checked for normality using the Shapiro–Wilk test. The primary endpoint was analysed in a confirmatory manner by applying an unpaired *t* test and comparing the change of the reactive hyperaemia index (RHI) at 3 months and at the study onset between the two groups. For changes within groups, the paired *t* test was employed. If data were not normally distributed, Wilcoxon matched pairs signed rank test was used. For evaluation of 7-day BP changes within a group, the data were analysed by using a repeated measures ANOVA test. If data were not normally distributed, the Friedman test was applied. Differences between parallel groups were evaluated by using an unpaired *t* test. If the differences were not normally distributed, the Mann–Whitney *U* test was applied. All statistical tests were performed as two-sided. Generally, a significance level of 0.05 was used.

## Results

The presentation of the efficacy results during phase I and of the safety results during phases I and II are based on the intention-to-treat (ITT) analysis. The efficacy results from the open follow-up phase II are based on per-protocol (PP) analysis. All results of the ITT analysis were confirmed by the PP analysis.

**Fig. 2:**
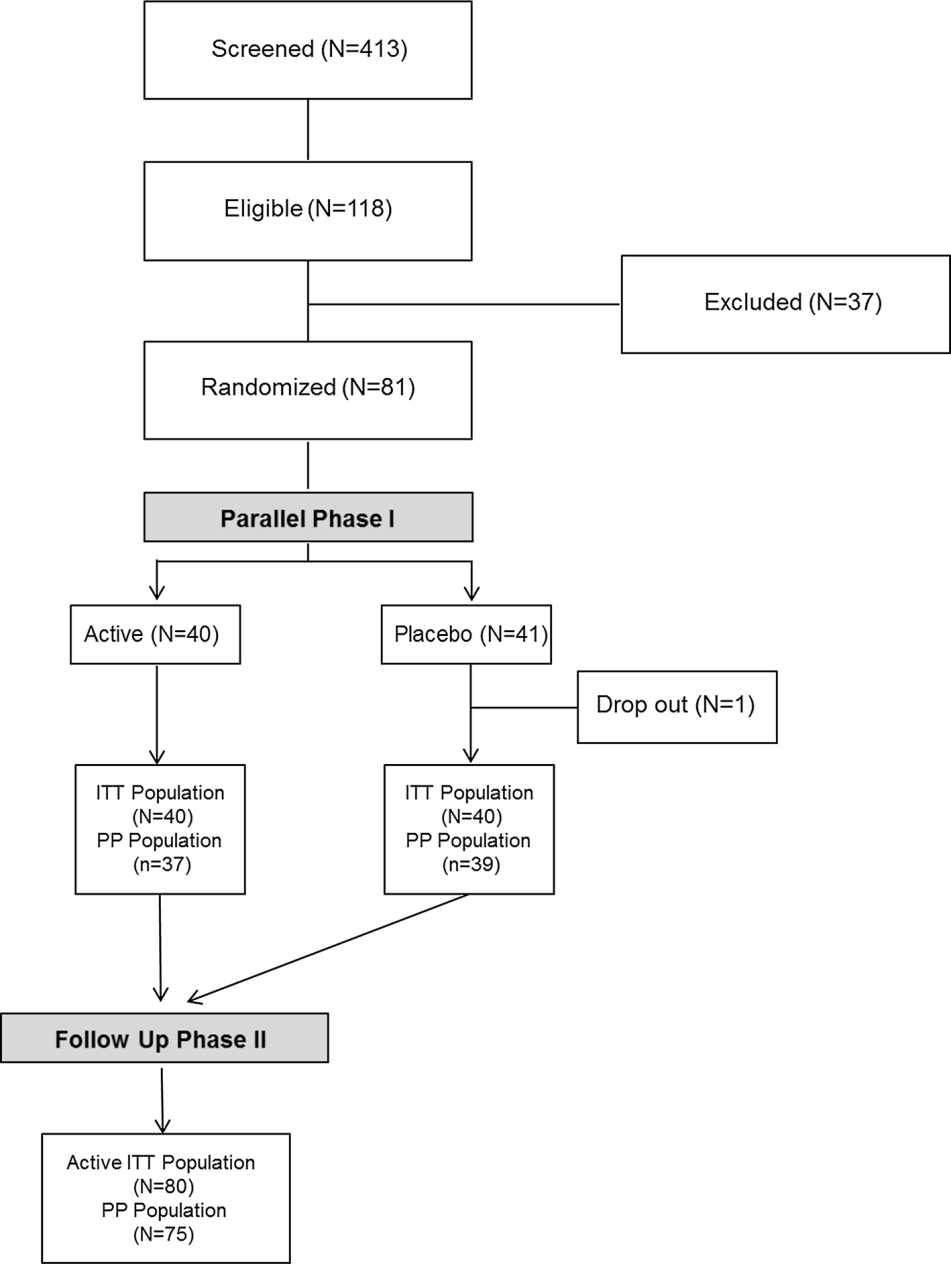
Subjects

*Subjects* From 81 enrolled subjects with slightly to moderately elevated BP, a total of 80 completed the entire study protocol resulting in 40 subjects for each group (Fig. 2). The compliance regarding the intake of study products for both study phases was very good in both groups. At baseline, nutritional status (0 = healthy, balanced nutrition, 100 = unbalanced nutrition) and individual stress level [46] (20 items in 4 categories resulting in 0 = no stress to 100 = maximum stress) were assessed by means of questionnaires (Table 1).

**Table 1:** Baseline characteristics of both groups

	Intervention	Placebo	*p*
*n*	40	40	
Age (years)	53.23 ± 6.41	54.30 ± 5.14	0.494^a^
Weight (kg)	78.45 ± 12.04	74.75 ± 12.21	0.176^b^
Height (m)	1.74 ± 0.10	1.73 ± 0.09	0.544^b^
BMI (kg/m^2^)	25.74 ± 0.40	24.83 ± 0.44	0.133^b^
Nutrition (score)	49.55 ± 8.11	48.03 ± 10.40	0.467^b^
Stress level (score)	34.00 ± 14.64	29.31 ± 17.29	0.194^b^

Values are mean ± SD

^a^Mann–Whitney *U* test, ^b^unpaired *t* test

**Fig. 3:**
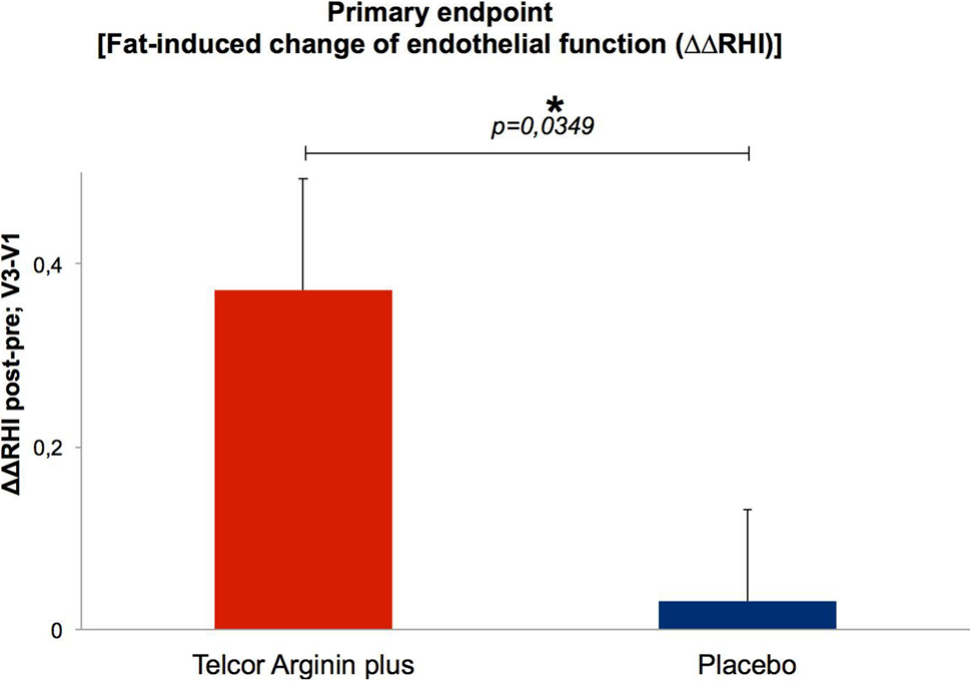
Improvement in endothelial function brought about by l-arginine and B vitamins. Values are mean ± SEM; unpaired t-test, ITT population

*Changes in ΔΔRHI* At baseline, no differences were detected between the two groups (*p* = 0.316). After 3 months, the primary study endpoint (changes in RHI post–pre, visit 3–visit 1; active compared to placebo, ΔΔRHI) reached statistical significance with a value of *p* = 0.0349. While in the active group ΔΔRHI increased significantly, almost no change could be detected in the placebo group (Fig. 3). Before and after the fatty load ΔΔRHI was increased in the intervention group by 0.371 ± 0.122, while in the placebo group ΔΔRHI increased by 0.031 ± 0.100. During visits 3 and 5, the significant increase in ΔΔRHI (*p* = 0.001) was confirmed in the intervention group, while no significant differences were found for the placebo group (*p* = 0.758).

*Blood pressure* During the first 3 months of supplementation, the systolic BP decreased significantly in both groups. In the placebo group, the decrease stagnated between visits 2 and 3 (systolic placebo V1: 138.0 ± 5.39 mmHg; V2: 133.2 ± 9.24 mmHg; V3: 133.0 ± 10.13 mmHg), whereas in the intervention group systolic values decreased further, similar to the decrease seen between visits 1 and 2 (systolic BP V1: 138.7 ± 5.11 mmHg; V2: 136.0 ± 7.94; V3: 133.8 ± 6.99 mmHg). The continuous decrease in the active group may have indicated substance effects, which was supported by the further analysis of the active group over the next 3 months of supplementation. Between visits 1 and 5, the fall in systolic BP was highly significant (*p* < 0.0001) so that values of 132.5 ± 7.22 mmHg were attained, although no significant inter-group differences were evident.

In the active group, there was a tendential decrease in diastolic BP from 87.7 ± 6.59 to 85.8 ± 6.79 mmHg, *p* = 0.061). Compared to placebo, the decrease was more pronounced in the active group (Δ diastolic BP V2–V1 placebo: −0.1 ± 5.42; intervention: −1.9 ± 4.27; *p* = 0.103). The results were further substantiated by the PP analysis: in the intervention group, diastolic values decreased significantly already after 3 months of supplementation (*p* = 0.048). In comparison with placebo, a trend indicating a reduction in diastolic BP was observed (*p* = 0.093). Over the ensuing 6 months of supplementation, the decrease in diastolic values in the intervention group then reached statistical significance (V1: 87.61 ± 6.775 mmHg; V3: 85.1 ± 6.966 mmHg; V5: 84.08 ± 7.354; *p* = 0.002), which confirms the positive influence of the dietary intervention on BP. The subgroup analysis revealed that the antihypertensive effect of the intervention was particularly evident in men where the decrease in diastolic BP was significant between the groups (*p* = 0.039).

**Fig. 4:**
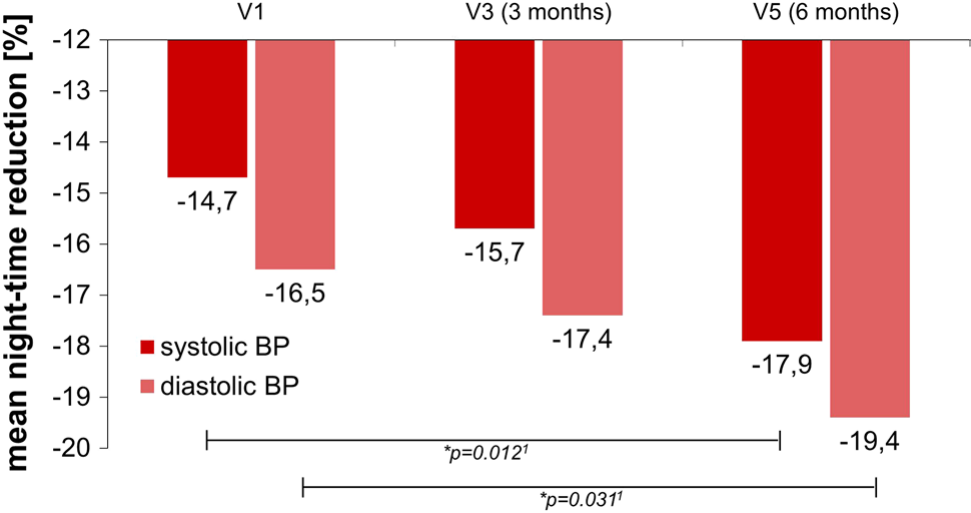
Enhanced night-time BP reduction (nocturnal dipping) after long-term dietary intervention in the intervention group. ^1^rmANOVA, ITT population

*Nocturnal BP dipping* Within the first 3 months of intervention, there was a tendential increase in the magnitude of systolic nocturnal dipping in the intervention group compared to the placebo group. A similar trend was seen for the diastolic nocturnal dipping. This effect in the intervention group during the first 3 months of supplementation was further reinforced during the subsequent 3 months of supplementation, when the nocturnal dipping of systolic (*p* = 0.012) and diastolic BP (*p* = 0.031) reached significance (Fig. 4).

**Fig. 5:**
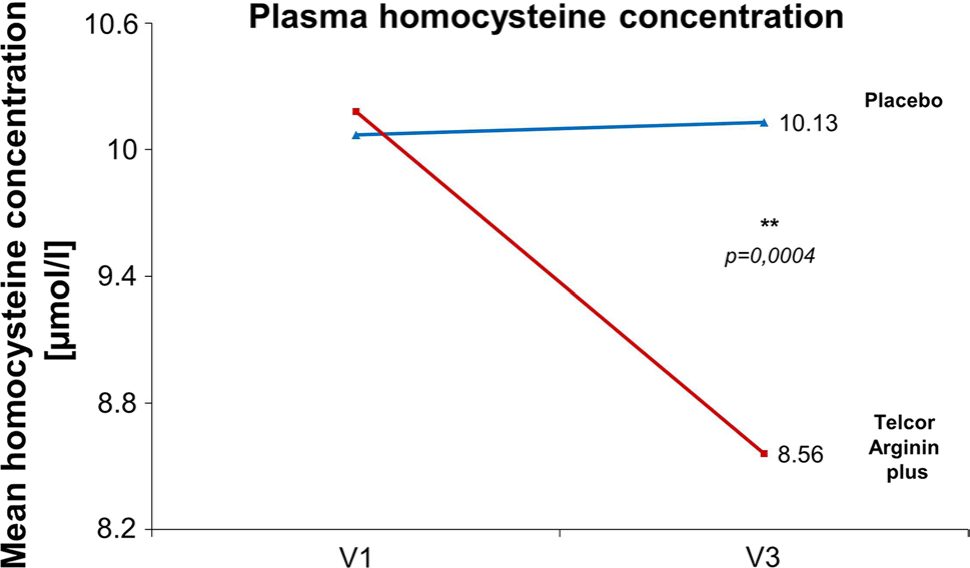
Reduction in homocysteine after 3 months. Mann–Whitney *U* test, ITT population

*Homocysteine blood concentration* After 3 months of supplementation, the homocysteine blood concentrations were significantly decreased from 10.2 ± 4.2 to 8.56 ± 2.04 µmol/l in the intervention group (*p* = 0.0001), whereas the values remained stable in the placebo group. The difference between the groups was statistically significant (*p* = 0.0004) with reductions amounting to +0.05 ± 1.29 µmol/l in the placebo group and −1.61 ± 3.34 µmol/l in the intervention group (Fig. 5).

In the intervention group, the reduction in homocysteine concentration was retained over the whole trial period. In the placebo group, when intervention was reverted to during the open phase, there was also a significant reduction by −1.41 ± 1.53 μmol/l compared to the levels measured after 3 months of placebo intervention.

*Safety and tolerability* All recorded adverse events (AE) were non-serious, and no difference in the frequency of AE could be detected between the groups. During the blinded parallel phase, in the placebo group, a total of 20 (48.8%) subjects with AE were recorded compared to 19 (47.5%) in the TAP group [31 (38.8%) of *n* = 80 during the follow-up]. Three participants assessed the AE (gastrointestinal tract disturbance; short time sleeping disturbances) as possibly related to TAP; however, the investigators judged the causal relationship as unlikely in all cases. There were no clinically relevant laboratory parameter changes in either group. Additional metabolic or inflammation parameters such as serum lipids or high-sensitivity C-reactive protein (not shown) were not influenced during the trial period and strongly support therapeutic safety. In both groups, most of the participants rated the tolerability with the maximum score of good. The findings confirmed the excellent safety profile of the investigational product.

## Discussion

This trial confirms the efficacy, safety and tolerability of a dietary intervention combining l-arginine and B vitamins for the purposes of improving cardiovascular health. The primary efficacy analysis demonstrates a statistically significant superiority of the l-arginine and B vitamin combination over placebo in improving and restoring the impaired endothelial function after fat loading in a group of subjects with slightly to moderately elevated BP.

The significant improvement in endothelial function in the intervention group indicates that l-arginine in combination with B vitamins is able to improve vascular function even after a strong dysfunctional trigger such as a fatty meal challenge. This effect was fully maintained over the entire trial duration of 6 months which demonstrates a long-lasting effect of the diet on the improvement in endothelial function.

All three main parameters and risk factors for cardiovascular disease, namely endothelial function, BP and homocysteine levels, were positively influenced by the nutrients. The positive effects were achieved by a daily supplementation of only 2.4 g of l-arginine in combination with small amounts of B vitamins. The results obtained suggest that the nutritional combination of l-arginine and B vitamins can successfully be employed to control cardiovascular risk factors, to improve endothelial function and to develop a dietary intervention of the early stages of atherosclerosis, stages which are associated with mild hypertension and hyperhomocysteinemia as well as moderately to slightly enhanced metabolic stress as defined by enhanced superoxide anion radical formation.

The findings from the current trial are consistent with numerous recent clinical studies, systematic reviews and meta-analyses of relevant clinical data in this field [1, 4, 5, 17, 22–24]. They demonstrate that a dietetic approach can successfully be employed to address ED at a very early stage of cardiovascular disease, which may therefore help to prevent progression and degenerative changes and lower the risk of severe complications.

The positive effect of l-arginine on impaired endothelial function has previously been demonstrated in volunteers after acute administration of 2.5 g of this amino acid [37]. Endothelial function induced by a fatty meal was determined by flow-mediated dilatation 1 h postprandial. The amino acids l-phenylalanine and l-leucine, however, did not prevent lipemic ED. In addition, l-arginine given alone without B vitamins had no acute effect on baseline flow-mediated dilatation. Thus, this study confirms and extends these findings by demonstrating the long-term efficacy of l-arginine in combination with vitamin B_6_, folic acid and vitamin B_12_ in preserving endothelial function in a population of subjects with slightly or moderately elevated BP. Comparable effects of l-arginine were demonstrated in numerous other clinical studies. The available findings were the subject of a systematic review with a meta-analysis published by Bai et al. [22] who demonstrated the efficacy of l-arginine in restoring endothelial function. The authors of this meta-analysis concluded that the studies show a normalization of endothelial function only in subjects who had substantially impaired vascular health. Sydow and Böger also discussed this topic in detail, explaining why l-arginine in contrast to B vitamins given alone can counteract ED in hyperhomocysteinemia [47]. However, until now we are not aware of any studies demonstrating efficacy of a low-dose combination of the cardiovascular nutrients l-arginine, vitamin B_6_, folic acid and vitamin B_12_ in restoring endothelial function over a period of 6 months.

l-arginine is a conditionally essential amino acid in the human diet [1, 2], and the most common dietary sources of l-arginine are meat, poultry and fish. However, individuals with poor nutrition or under conditions of increasing age and certain physical conditions, e.g. ED, hypertension or diabetes, may be advised to supplement l-arginine in sufficient amounts. In a recent study, Ganz and colleagues demonstrated that serum l-arginine levels are lower in individuals with type 2 diabetes mellitus compared to controls, and that l-arginine levels were inversely related to HgbA1c [18]. l-arginine, which acts by many different signal transduction pathways, is the most important precursor for the synthesis of nitric oxide (NO), a key signalling molecule. Reduced bioavailability of vasoprotective NO plays an essential role in cardiovascular pathologies and metabolic diseases. NO synthases (NOS) oxidize the NO precursor l-arginine to NO and citrulline [1, 2].

**Fig. 6:**
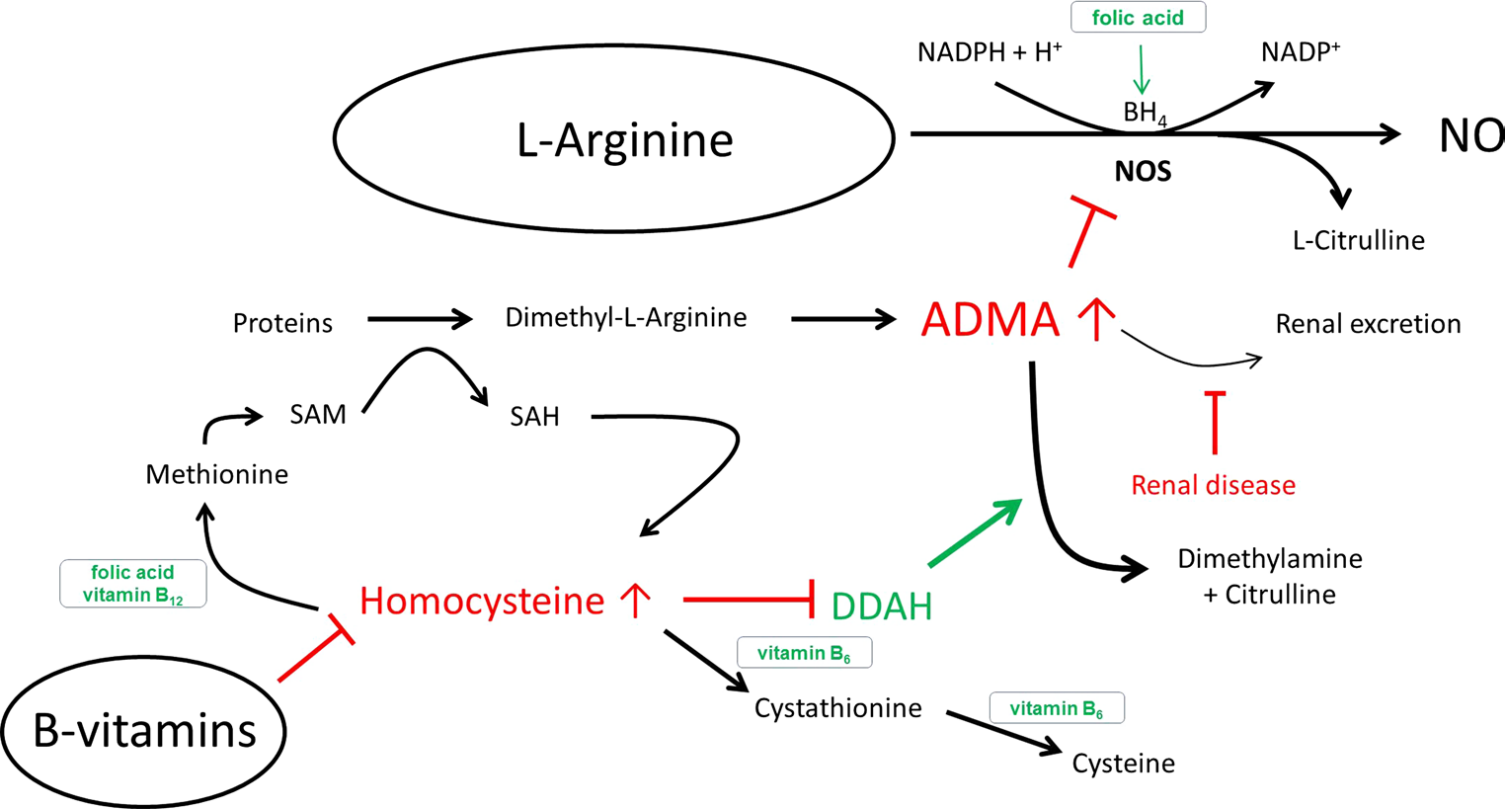
Synergistic effects of l-arginine and B vitamins

Tetrahydrobiopterin (BH4) is a cofactor for several important enzyme systems including the NOS isozymes [5]. Experimental and clinical studies implicate BH4 as a key regulator of NO formation from l-arginine by eNOS. As such, BH4 has to be considered a key factor in determining cardiovascular health and disease [48–51]. The augmentation of endothelial BH4 levels by folic acid has been shown to enhance NO formation (for review see [5]). Folates are able to increase gene and enzyme expression as well as NO formation by acting as cofactors or recycling agents for BH4. They increase bioavailability and bioactivity of BH4 and prevent premature degradation with uncoupling of the enzyme to produce superoxide anion radicals instead of NO [51–53]. Whereas folic acid directly affects NO formation from l-arginine, the other B vitamins can support several additional metabolic processes that safeguard NO formation from its endogenous substrate [51, 52]. The most important metabolic pathways that are targeted by B vitamin supplementation have been identified [5, 47, 51, 52] and have been shown to be operational under administration of l-arginine and these micronutrients (Fig. 6).

In addition, the synergistic effect of B vitamins should improve the NO-mediated responses induced by l-arginine. Since superoxide anion radicals can be generated upon uncoupling of NO synthesis from its endogenous precursor l-arginine through transfer of electrons to oxygen instead, the addition of antioxidative B vitamins can safeguard the necessary coupling reaction and therefore prevent oxidative or nitrosative stress associated with high homocysteine and ADMA levels.

In the current clinical study, it could be confirmed that even relatively low nutritional amounts of l-arginine, if combined with small amounts of vitamins B_6_, folic acid and B_12_, can effectively prevent ED induced by a fat load. Most commonly, in clinical studies endothelial function is measured by using flow-mediated dilatation (FMD) of the brachial artery [54–56]. However, FMD is known to be highly user dependent since it requires specially trained personnel [57]. In this study, we employed the non-invasive, easy-to-use and user-independent pulse amplitude tonometry (EndoPAT) which has already extensively been used in several studies assessing endothelial function [57–61]. In addition, this method adequately depicts the overall health of the endothelium [38, 39] which corresponds with the indication of the nutritional product and which emphasizes that the endpoint is not only validated, but also clinically important.

In numerous studies, endothelial function is measured by brachial artery flow-mediated dilatation (FMD) 3 h after a fat load [37, 62, 63]. However, while FMD measures the endothelial function of the macrovascular system, the EndoPAT technique measures the endothelial function in the microvascular system. Since it was shown that, compared to the macrovascular system, the reaction of the microvascular system to postprandial challenge is faster [64], the measurement point was set to 1 h following a fat load. Since a comparable combined metabolic and endothelial function effect is not known from the individual nutrients at the given doses, synergism of their combination can be reasonably assumed resulting in a long-lasting and statistically significant vascular effect which was a major finding of this study. The contribution of B vitamins to the BP-lowering effects of l-arginine by potentiating the response to the amino acid is consistent with the literature [5, 47, 51, 52].

The restoration and maintenance of endothelial function enable NO-induced vasodilatation which is supported by a significant blood pressure reduction as demonstrated by a meta-analysis of clinical studies where active treatment was given in the form of l-arginine [23]. Vasodilatation by NO can lower BP by simultaneously increasing blood flow [1, 2].

Home blood pressure monitoring was included to overcome bias in office-based BP readings such as digit preference, observer bias [65] or the “white coat effect”. Compared to office-based BP measurements, home BP monitoring holds superior reproducibility and, in addition, predictive ability in terms of hypertensive target organ damage and prognosis of cardiovascular disease [66].

In the current trial, mild to moderate hypertension was chosen as an inclusion criterion. Due to the small BP elevation level at baseline, however, the margin for BP lowering itself was expected to be limited. Nevertheless, BP-decreasing effects were observed for systolic, diastolic and night-time BP to differing extents. During the first 3 months, the systolic BP decreased significantly in both groups, but in the placebo group the decrease stagnated between visits 2 and 3 while in the active group systolic values continued to decrease, thus substantiating the efficacy of the nutritional intervention. The decrease in diastolic BP was more pronounced in the intervention group during the blind phase, but the inter-group difference did not reach statistical significance. Subgroup analysis revealed that the antihypertensive effect of the intervention was particularly evident in men where a decrease in diastolic BP was seen with a significant difference between the groups (*p* = 0.0387).

The BP-lowering potential of the test product was also reflected in the night-time BP reduction (i.e. nocturnal dipping). Studies have shown that attenuated nocturnal blood pressure dipping is a better predictor for cardiovascular morbidity and mortality than resting BP measurements (for review see [67]). Moreover, attenuated nocturnal dipping is associated with elevated levels of molecules related to ED and atherosclerosis [68]. The continuous improvement in nocturnal BP reduction observed over the entire study duration should be considered clinically relevant since it was associated with positive effects on endothelial function.

A limitation of the study was the single-centre design since, compared to multi-centre trials, single-centre trials can show a small increase in effect [69]. In our trial, this limitation is predominantly compensated by the following facts: (1) the RHI provided objective results; (2) the difference in effect between the active intervention and the comparator was pronounced; while in the active group ΔΔRHI increased significantly, almost no change could be detected in the placebo group; (3) all three main parameters (endothelial function, BP and homocysteine levels) have displayed positive effects.

An important aspect is that the sample also corresponds to the typical field of application of a dietetic preparation. This supports the high external validity of the trial because its design is representative for routine practice where the choice would be to offer a dietary product alone or as a dietary add-on to drug treatment.

In an earlier large non-interventional study (*n* = 477) with the test product, the systolic BP decreased by 11.4 from 147.3 to 135.9 mmHg, and the diastolic BP fell by 6.6 from 88.45 to 81.9 mmHg [70]. However, in that study the treatment duration was up to 27.4 months and patients were characterized by a higher mean baseline BP so that the scope for decreasing BP was larger. These findings seem particularly relevant considering the low BP reductions that can be achieved with conventional antihypertensive drugs. A significant reduction in systolic BP by more than 6 mmHg and diastolic BP by more than 1.5 mmHg is considered to be highly relevant [71]. In a recent review, based on more than 600,000 participants even modest changes in blood pressure were evaluated as being clinically significant. For example, a 5-mmHg reduction in systolic blood pressure was associated with a 14% reduction in stroke risk and a 9% reduction in coronary heart disease risk [72, 73]. Thus, it is justified that the positive effects of the test preparation on BP in our mildly hypertensive patients are interpreted as clinically relevant.

Hyperhomocysteinemia may constitute an independent risk factor for the development and progression of cardiovascular disease associated with ED. Hyperhomocysteinemia and vitamin B supplementation have been discussed in the context of the opposing effects of l-arginine and ADMA on endothelial function, atherosclerosis and cardiovascular disease risk factors. Homocysteine has been shown to inhibit dimethylarginine dimethylaminohydrolase (DDAH) which is responsible for ADMA degradation, e.g. by oxidative inactivation of the active-site cysteine residue of DDAH, by forming reactive intermediates and by enhancing oxidative stress [47].

Since a lowering effect of vitamin B supplementation on homocysteine concentration is the subject of some debate [47], a need exists for interventional trials investigating the effects of B vitamins alone and combined with l-arginine at various dosages. Also, different populations need to be studied, as pointed out in a recent review by Lundberg and colleagues [74], in order to come to sound conclusions about how these agents might be recommended to bring about successful effects. The current trial fills this gap since results were obtained under randomized, controlled conditions with subjects aged 40–65 years with slightly to moderately elevated BP. After 6 months of supplementation, homocysteine was significantly decreased from 10.2 to 8.5 µmol/l in the active group, whereas the values remained stable in the placebo group. The difference between the groups was statistically significant.

The extent to which the reduction in homocysteine contributed to the improvement in impaired endothelial function in this trial can, however, not be estimated. Since higher homocysteine concentrations are associated with enhanced ADMA and superoxide anion radical levels [52], a maximal lowering of blood homocysteine levels would seem to be a rational goal. The marked reduction in homocysteine levels seen in this trial can be attributed to the synergistically acting B vitamins, and particularly to folic acid.

Dietary management with l-arginine in combination with B vitamins resulted in a significant improvement in endothelial function, which may also be mediated by the endothelial cell glycocalyx, a novel important structure in the function and regulation of the endothelium. It has been shown by several authors that the glycocalyx is damaged and may be partially destroyed by reactive oxygen species (ROS) [75–77]. A disturbed balance of the NO/ROS system in our patient cohort may have led to a disturbance of the glycocalyx resulting in impaired endothelial cell function. Conversely, the nutritional intervention with l-arginine may have influenced the endothelial cell glycocalyx and resulted in a restauration of this important endothelial structure. Recently, novel tools to assess the glycocalyx in patients have been introduced [78]. Using these novel methods, we will investigate the influence of l-arginine on the glycocalyx and test the hypothesis that l-arginine regulates and improves the structure and function of the endothelial glycocalyx.

Epidemiologic data support the usefulness of dietary supplementation with l-arginine and B vitamins. As such, the intake of nuts and the Mediterranean diet in general are rich sources of l-arginine and B vitamins, and all have been proved to significantly reduce cardiovascular morbidity and mortality [79–82].

The findings of the current clinical trial confirm the excellent safety profile found in an earlier prospective, multi-centre, non-interventional dietetic study which was also performed with the preparation tested here (*n* = 484).

The dietetic intervention was proved to be effective, well tolerated and safe. The result was achieved through a daily supplementation of 2.4 g l-arginine in combination with B vitamins, confirming the practical relevance of the synergism between these nutrients when used in small amounts. The dietary intervention can be considered a physiological alternative and addition to conventional treatment of cardiovascular disorders which addresses the underlying pathophysiology. As such, and particularly for subjects with mildly to moderately enhanced BP and/or early stages of atherosclerosis where impaired endothelial function is evident, the innovative nutritional combination of l-arginine and B vitamins might successfully be used to complement the existing therapeutic repertoire of methods so that cardiovascular health can be maintained, improved or even restored.
